# Combining genetic association study designs: a GWAS case study

**DOI:** 10.3389/fgene.2013.00186

**Published:** 2013-09-27

**Authors:** Janice L. Estus, Family Investigation of Nephropathy and Diabetes Research Group, David W. Fardo

**Affiliations:** ^1^Department of Biostatistics, University of KentuckyLexington, KY, USA; ^2^Genetic Analysis and Data Coordinating Center, Family Investigation of Nephropathy and Diabetes, Case Western Reserve UniversityCleveland, OH, USA

**Keywords:** genome-wide association, combined study design, family-based association analysis, case-control study, diabetic nephropathies

## Abstract

Genome-wide association studies (GWAS) explore the relationship between genome variability and disease susceptibility with either population- or family-based data. Here, we have evaluated the utility of combining population- and family-based statistical association tests and have proposed a method for reducing the burden of multiple testing. Unrelated singleton and parent-offspring trio cases and controls from the Genetics of Kidneys in Diabetes (GoKinD) study were analyzed for genetic association with diabetic nephropathy (DN) in type 1 diabetics (T1D). The Cochran-Armitage test for trend and the family-based association test were employed using either unrelated cases and controls or trios, respectively. In addition to combining single nucleotide polymorphism (SNP) *p*-values across these tests via Fisher's method, we employed a novel screening approach to rank SNPs based on conditional power for more efficient testing. Using either the population-based or family-based subset alone predictably limited resolution to detect DN SNPs. For 384,197 SNPs passing quality control (QC), none achieved strict genome-wide significance (1.4 × 10^−7^) using 1171 singletons (577/594 cases/controls) or 1738 pooled singletons and offspring probands (841/897). Similarly, none of the 352,004 SNPs passing QC in 567 family trios (264/303 case/control proband trios) reached genome-wide significance. Testing the top 10 SNPs ranked using aggregated conditional power resulted in two SNPs reaching genome-wide significance, rs11645147 on chromosome 16 (*p* = 1.74 × 10^−4^ < 0.05/10 = 0.005) and rs7866522 on chromosome 9 (*p* = 0.0033). Efficient usage of mixed designs incorporating both unrelated and family-based data may help to uncover associations otherwise difficult to detect in the presence of massive multiple testing corrections. Capitalizing on the strengths of both types while using screening approaches may be useful especially in light of large-scale, next-generation sequencing and rare variant studies.

## Introduction

The successes and failures of genome-wide association studies (GWAS) have made for both interesting scientific dialog and the development of innovative statistical methodologies. While debate continues around reasons for the so-called missing heritability of GWAS, the sheer number of replicable genetic associations discovered using this approach is unarguable (Hindorff et al., 2013). Next-generation sequencing has taken the baton (or at least begun its own race) to continue the search for genetic association with complex disease outcomes. Many unique analytical issues have arisen with sequencing data, but two paramount themes of concern, in particular, persist regardless of the assay technology—quality control (QC) and study design. Here, we examine the latter in the context of the Genetics of Kidneys in Diabetes (GoKinD) study, a GWAS comprising one subset of unrelated subjects and another of mother-father-proband trios.

The relative merits of a genetic association study being designed around either families or unrelated subjects, most often cases and controls, has been addressed previously (Fardo et al., 2012). Briefly, case-control studies are generally considered easier to implement, less costly and more powerful than studies incorporating related subjects. Family-based studies on the other hand are robust to the discovery of spurious association due to unresolved population substructure and also provide more textured information such as improved haplotype resolution, Mendelian error checking and the ability to test for imprinting effects. This obviously oversimplifies the comparison of two very broad classes of designs—in this work we are concerned with implications of combining the two rather than simply choosing one or the other.

Many genetic association studies spawn from existing cohorts that either had previously employed linkage analysis with pedigree recruitment (Clerget-Darpoux and Elston, 2007) or had initially not explored genetic risk factors. Studies in these scenarios can then quite naturally comprise both unrelated subjects and families. Because this is not uncommon, there are many statistical methodologies that have been developed to combine information from unrelated cases and controls with family pedigrees, and several of these have been compared via simulation (Fardo et al., 2011). Our focus here is not to again compare distinct methodologies across a simulation study but rather to compare simple, easily implementable approaches in handling the unrelated and family subsets from the GoKinD study.

The GoKinD study aimed to identify genes associated with diabetic nephropathy (DN) in type 1 diabetics (T1D). T1D patient probands were screened to identify cases with kidney disease and controls with normal renal status despite long-term diabetes. When possible, both parents of the proband were enrolled to form family trios and DNA was collected for all T1D patients and participating parents (Mueller et al., 2006). In the original GWAS, Pezzolesi et al. (2009) combined all GoKinD cases and controls (unrelated singletons and trio offspring) to test for genetic association with DN. No single nucleotide polymorphism (SNP) reached genome-wide significance but several loci were “suggestive” (*p* < 1 × 10^−5^). This strategy of combining the offspring from trios, or, more generally, a randomly selected non-founder from a pedigree, with the unrelated cases and controls has been common practice (Infante-Rivard et al., 2009).

Here, we propose a simple, intuitive, and straightforwardly implementable strategy to combine association metrics from unrelateds and families while providing a working solution to the multiple testing problem when these types of data are available. The main goal of this work, however, is two-fold: to thoroughly examine the differences between first-pass approaches and those using all available information; and to make the case for using and developing methods for aggregation while suggesting a direction for this methodological research. Due to the nature of the study designs employed, the GoKinD study is an ideal dataset to present these comparisons. In what follows, we further describe the motivating GoKinD dataset and QC procedures employed, we outline the various methodological approaches explored including our initial suggestion of a combined screening and testing method, and finally we thoroughly compare results from the GoKinD study.

## Methods

### The GokinD study

#### Subjects

Detailed information regarding these data can be found in Mueller et al. (2006). Briefly, the GoKinD study comprises 1869 T1D patients with and without kidney impairment who were recruited through the George Washington University Biostatistical Center (GWU) and the Joslin Diabetes Center, section of Genetics and Epidemiology (JDC). Patients were 18–59 years old at the time of enrollment, received a T1D diagnosis before age 31 and had diabetes duration of more than 10 years in cases and more than 15 years in controls. DN cases were defined as either persistent proteinuria or end stage renal disease requiring dialysis or renal transplant. Controls were defined as having normal renal function and normal urine albumin. Of the 1285 unrelated singletons (664/621 DN cases/controls) and 584 mother-father-offspring trios (268/316 DN case/control offspring) recruited and genotyped, 1270 unrelated singletons (651/619 DN cases/controls) and 571 mother-father-offspring trios (266/305 DN case/control offspring) were released for analysis through dbGaP (Mailman et al., 2007; Pluzhnikov et al., 2010).

#### Quality control

We replicated the extensive and well-documented QC procedures conducted in the original GoKinD GWAS which employed the Affymetrix 5.0 500K SNP array (Pezzolesi et al., 2009). To maintain consistency, we repeated the entire QC pipeline with and without the addition of trio offspring cases and controls using the 469,094 SNPs provided by dbGaP. The former mirrors the original study that incorporated family data which allowed for additional Mendelian error QC filtering and the latter comprises the QC for the population-based subset within the proposed methodology and typical of case-control GWAS studies. Over 35,000 SNPs were removed due to the detection of 3 or more trios exhibiting a Mendelian error (Supplemental Table 2). Principal component analysis (PCA) was applied to both population-based subsets to minimize spurious associations due to population substructure by removing potential ethnic outliers (Price et al., 2006) (Supplemental Figure 2). More details on QC can be found in the Supplementary Materials.

### Statistical analysis

**Figure 1:**
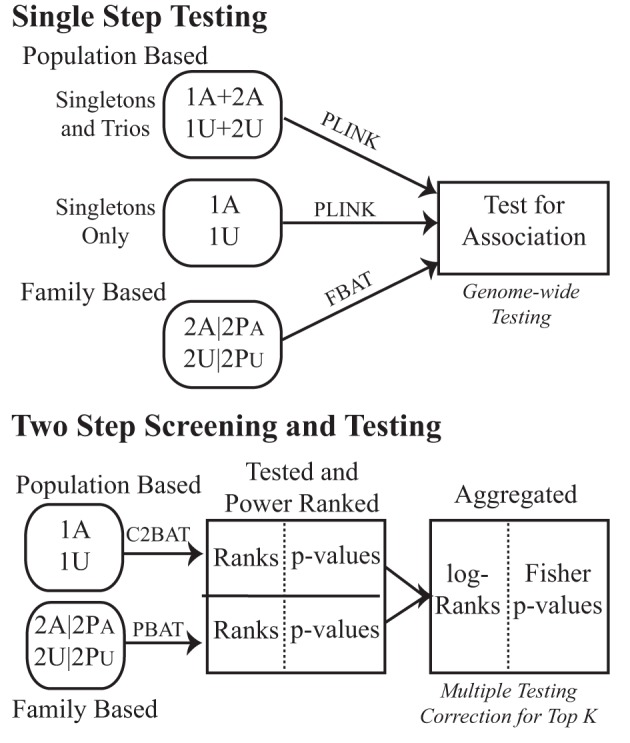
**Testing schematic for the GoKinD collection**. Subjects with type 1 diabetes with (affected) and without (unaffected) diabetic nephropathy were studied for genetic association. Population- and family-based subsets were either tested in a typical straight-forward single-step strategy or in a two-step combination strategy with conditional screening for power, association testing and subsequent combination of the two. To distinguish between the data subsets used, 1′ s indicate unrelateds and 2′ s are from the family samples. A denotes affected and U unaffected. The analytic methods used are indicated above the corresponding arrows.

We first compared the approach of separating subjects into subsets of unrelated population-based cases and controls (singletons) and family-based subjects (trios), against adding the trio offspring into a pooled unrelated subset, to analyze using common case-control statistics as in the initial analysis of Pezzolesi et al. (2009). We then implemented a two-step approach combining statistical tests across unrelated and family-based study designs (Figure 1).

#### Population-based association

Genetic association using the subset of unrelateds was examined using the Cochran-Armitage test for trend assuming an additive genetic mode of inheritance. The trend test was adjusted for sex and stratified by center using a Cochran-Mantel-Haenszel test as in the original GoKinD GWAS. These tests were conducted with and without the addition of offspring cases and controls in order to replicate the original findings and to use within the proposed framework, respectively (Figure 1; Singletons Only vs. Singletons and Trios). All analyses were conducted using the freely-available softwares PLINK (Purcell et al., 2007) and R (R Development Core Team, 2010).

#### Incorporation of trio parents

Along with adding resolution for QC, the addition of parents makes possible traditional family-based association testing (FBAT). FBATs were calculated using the FBAT package (Laird et al., 2000) assuming a DN prevalence within T1D of 30% (Krolewski et al., 1996; Steinke, 2009). Using true prevalence as an offset in the FBAT numerator is known to maximize power for the test in population samples (Whittaker and Lewis, 1998; Lunetta et al., 2000; Lange and Laird, 2002). Because ascertainment was not conditioned on DN status in GoKinD, this estimate should perform optimally.

#### Fisher's combined probability test

We adopted a simple procedure to combine test statistics across study designs. Fisher's method (Fisher, 1925), often used in meta analyses, is a commonly used approach to aggregate independent p-values. Here, our testing is done in two separate subsets, family trios and case-control singletons, which maintains the independence necessary to implement this test. There are other methodologies to combine *p*-values, and all of our work could be adapted straightforwardly to accommodate alternative choices.

#### Dealing with multiple comparisons

For the trio subset, offspring genotypes are treated as missing and then imputed assuming Mendelian transmissions from parental genotypes in order to provide information for screening that is completely independent of the actual family-based association test. That is, offspring genotypes are not used in the screening step so that they may be used in a completely independent testing step. SNPs with favorable configurations (i.e., enough allelic variation and informative families) will be ranked highly by virtue of providing more likelihood of finding an association that is present. More formally, the Van Steen algorithm (Van Steen et al., 2005) decomposes the joint data likelihood into two independent pieces [i.e., P(Y,X,S) = P(X|Y,S)P(Y,S), where Y is the offspring phenotype, X is the offspring genotype score (e.g., the count of minor alleles) and S are the sufficient statistics for offspring genotypes which are equal to the parental genotypes when available]. SNPs are screened based on information from P(Y,S), either from obtaining significance rankings from regression of Y on E(X|S) or from analytically calculating the conditional power for a SNP-phenotype pair; we employed the latter approach throughout. Note that E(X|S) is simply the expected offspring genotype score given the parental genotypes. These analyses were conducted using the freely-available PBAT software (Lange et al., 2004). The SNP rankings produced in this step use information that is completely independent of the offspring genotypes so that FBAT test statistics are orthogonal and do not require adjustment from the screening step. Thus, the top 10 SNPs, for example, can be tested with only a multiple testing adjustment for the 10 tests conducted. Extensions to the top K approach have been developed and could easily be employed (Ionita-Laza et al., 2007). The screening step is susceptible to effects of population stratification, but the testing step remains robust to spurious association.

C2BAT as proposed by McQueen et al. (http://rss.acs.unt.edu/Rdoc/library/pbatR/html/c2bat.html) and described by Sharma et al. (2012) was developed as the case-control analog to the Van Steen screening approach. Information from each SNP is split in order to screen for highly powered SNPs and then independently test for association. Similar to conditioning on the sufficient statistics for offspring genotypes, the random variables in the family-based testing framework, the margins of the affection-by-genotype contingency table are the appropriate sufficient statistics for the corresponding cell counts, which are the random variables in the population-based framework. Briefly, the C2BAT algorithm splits subjects from the contingency table into a non-informative table for screening and a testing table. These splits can be done to preferentially over-select minor homozygotes for the testing step. We employed the default selection of 75%, 50%, 25% minor homozygotes, heterozygotes, and major homozygotes to the testing table, respectively. The margins of the resulting testing table are used to randomly impute (under the null) cell counts, which are then combined with the non-informative table to rank SNPs. The testing table is then used to perform an orthogonal test for association for the highest ranking SNPs. We used the C2BAT version implemented in the pbatR package (Hoffmann and Lange, 2013).

To combine the rankings between the trio and case-control subsets, we averaged log-transformed rankings to come up with an aggregate ranking. The top 10 SNPs were then assessed for statistical significance at a lower multiple testing penalty (i.e., 0.05/10 = 0.005).

Note that our selection approach results in rankings equivalent to those from multiplying the rankings from both subsets. Importantly, this method is subjectively chosen and can likely be optimized in future research.

#### Methodological comparisons

Our primary methodology to combine information across study designs employs p-value aggregation, so we compare our approach to METAL (Willer et al., 2010), an efficient meta-analysis program that incorporates the disparate association information via sample size weights and effect directionality into an aggregate statistic. Briefly, each of the trio and singleton test *p*-values is converted into a *Z*-score and then weighted by the square root of the subset sample size to comprise a meta-analytic *Z*-score. In addition to meta-analytic methodologies, we also compared to an approach the aggregates data across the subsamples rather than the p-values (Zhang et al., 2009). The method proposed by Zhang et al. was chosen due to its implementation in the Genetic Association analysis Platform (GAP) and its superior performance in a previous comparison between other similar data aggregation methodologies (Fardo et al., 2011). The proposed score test comprises components from each subsample separately and is explained in detail in Zhang et al. (2009). Similar to the FBAT approach, we employ a phenotypic offset equal to the estimated prevalence of DN in T1D.

## Results

**Figure 2:**
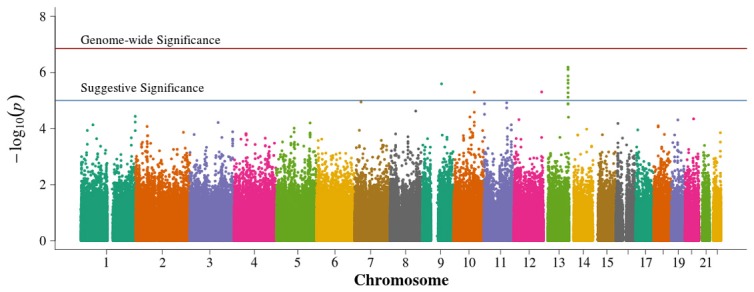
**Manhattan plot for population-based study with pooled singletons and trio probands**. Summary of genome-wide association scan results in the GoKinD population-based singletons and trios combined. The −log_10_*P*-values were calculated for SNP association with diabetic nephropathy among subjects with type 1 diabetes using the Cochran-Armitage test for trend for an additive genetic model adjusted for sex and stratified by center ascertainment using Cochran-Mantel-Haenszel method. The red horizontal line corresponds to genome-wide significance (*P*-value = 0.05/357,887 = 1.4 × 10^−7^). The blue horizontal line corresponds to suggestive significance (*P*-value = 1 × 10^−5^).

**Figure 3:**
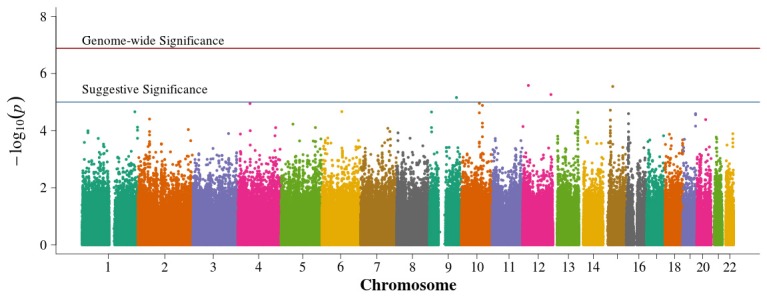
**Manhattan plot for population-based study with case and control singletons only**. Summary of genome-wide association scan results in the GoKinD cases and controls, singletons only. The −log_10_*P*-values were calculated for SNP association with diabetic nephropathy among subjects with type 1 diabetes using the Cochran-Armitage test for trend for an additive genetic model adjusted for sex and stratified by center ascertainment using Cochran-Mantel-Haenszel method. The red horizontal line corresponds to genome-wide significance (*P*-value = 0.05/384,094 = 1.3 × 10^−7^). The blue horizontal line corresponds to suggestive significance (*P*-value = 1 × 10^−5^).

In the population-based analyses, no SNP achieved Bonferroni adjusted genome-wide significance for association with DN in T1D (0.05/384,197 = 1.3 × 10^−7^). Areas of suggestive association noted in the pooled population-based analysis (Figure 2) are diminished in the singletons alone analysis (Figure 3). In the singleton alone subset, only four SNPs exceeded a suggestive *p*-value of 1 × 10^−5^.

**Figure 4:**
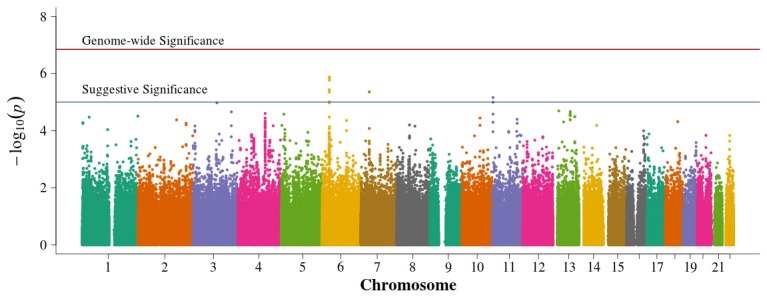
**Manhattan plot for family-based study**. Summary of genome-wide association scan results in the GoKinD cases and controls family-based trios and duo parent/offspring pairs. The −log_10_*P*-values were calculated for SNP association with diabetic nephropathy among subjects with type 1 diabetes using the generalized FBAT method with an offset of 0.3 (the prevalence of diabetic nephropathy in type 1 diabetics). The red horizontal line corresponds to genome-wide significance (*P*-value = 0.05/351,951 = 1.4 × 10^−7^). The blue horizontal line corresponds to suggestive significance (*P*-value = 1 × 10^−5^).

In the family-based analysis, no SNP achieved genome-wide significance using an FBAT statistic (Figure 4). Suggestive areas of associations in chromosome 11p in the *CARS* gene (cysteinyl-tRNA synthetase) were similar to results from Pezzolesi et al. (2009). New areas of interest in chromosome 6p within the major histocompatibility complexes (MHC) class II and III and in chromosome 7p are noted (Supplemental Table 3). The 13q chromosomal peak reported by Pezzolesi et al. (2009) was not observed.

**Figure 5:**
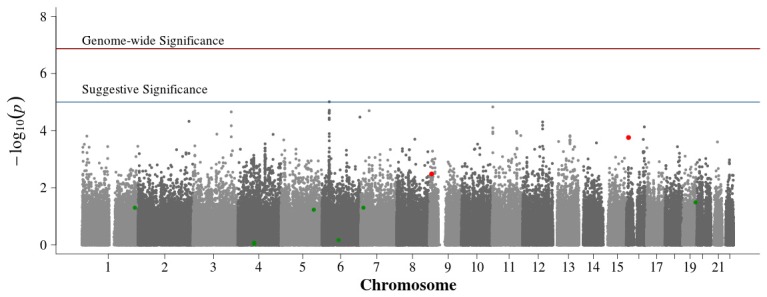
**Manhattan plot for Fisher's combined probability of population- and family-based studies**. Summary of genome-wide association scan results in the GoKinD collection of combined probability of the population-based and family-based *P*-values. The −log_10_*P*-values were calculated for SNP association with diabetic nephropathy among subjects with type 1 diabetes by combining each study *P*-values using Fisher's combined probability method. Power ranking was obtained using conditional mean model for family-based data and data partitioning for population-based cases and controls. Rankings were obtained for each subset and then log transformed and summed. The top ten ranked SNPs were tested; the two SNPs significant at 0.05/10 = 0.005 are indicated in red, while the other eight are in green. The red horizontal line corresponds to genome-wide significance (*P*-value = 0.05/374,042 = 1.3 × 10^−7^). The blue horizontal line corresponds to suggestive significance (*P*-value = 1 × 10^−5^).

No SNPs achieved significance using Fisher's combined probability method without the benefit of Van Steen-type screening approaches (Figure 5). The SNPs of suggestive significance in the population-based singleton only and pooled singleton and trio proband analysis were diminished by the addition of family-based information, suggesting potential population structure correction. Compared to the family-based subset, associations remained similar in other regions.

**Figure 6:**
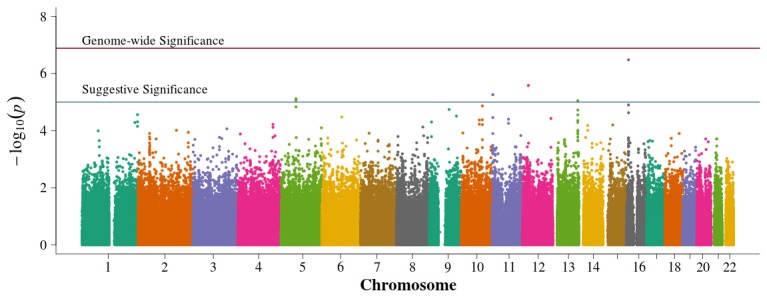
**Manhattan plot for METAL**. Summary of genome-wide association scan results in the GoKinD collection of the meta-analyzed population-based and family-based P-values. The −log_10_*P*-values were calculated for SNP association with diabetic nephropathy among subjects with type 1 diabetes by combining each study *P*-value using the METAL sample size method. The red horizontal line corresponds to genome-wide significance (*P*-value = 0.05/385,830 = 1.3 × 10^−7^). The blue horizontal line corresponds to suggestive significance (*P*-value = 1 × 10^−5^).

**Figure 7:**
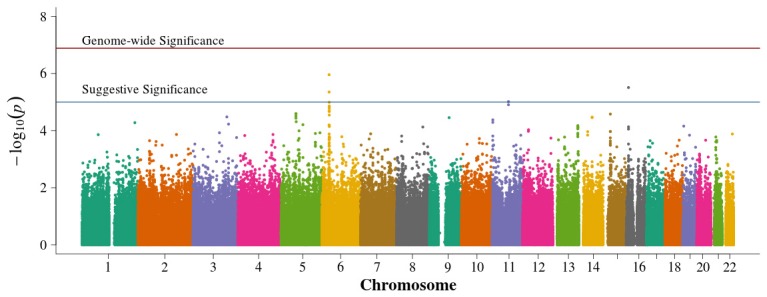
**Manhattan plot for GAP**. Summary of genome-wide association scan results in the GoKinD collection of combined population-based and family-based data. The −log_10_*P*-values were calculated for SNP association with diabetic nephropathy using the method of Zhang et al. (2009). The red horizontal line corresponds to genome-wide significance (*P*-value = 0.05/386,822 = 1.3 × 10^−7^). The blue horizontal line corresponds to suggestive significance (*P*-value = 1 × 10^−5^).

There were no genome-wide significant SNPs from either METAL or GAP, although six and four SNPs reach the suggestive significance level for METAL (Figure 6) and GAP (Figure 7), respectively. Three of these variants were not identified using other approaches. GAP analysis supports the chromosome 6p finding from the FBAT. This region harbors multiple genome-wide significant SNPs when employing either FBAT or GAP without the optimal phenotypic offset (not shown) and may actually be testing for T1D associations rather than those from DN within a TID population since, without the offset, the analysis reduces to a traditional, affecteds-only TDT.

Selection of the top 10 ranked SNPs from screening approaches combined across the unrelated and trio subsets and testing with Fisher's test resulted in two SNPs achieving genome-wide significance (*p* = 0.05/10 = 0.005; Table 1, Supplemental Table 3, Figure 5). SNP rs7866522 on chromosome 9p (*p*-value = 0.0033) is contained in the protein tyrosine phosphatase, receptor, D gene (PTPRD). Members of the protein tyrosine phosphatase family are known to be signaling molecules which regulate processes such as cell growth, differentiation, mitotic cycle, and oncogenic transformation (Wheeler et al., 2007). This region has been in identified in type 2 diabetic risk genome-wide studies (Tsai et al., 2010; Below et al., 2011; Chang et al., 2012) potentially related to glucose homeostasis and insulin sensitivity (Ren et al., 1998; Chagnon et al., 2006). SNP rs11645147 on chromosome 16p (*p*-value = 0.00017) is located in proximity to the glutamate receptor, ionotropic, N-methyl D-aspartate 2A gene (GRIN2A).

**Table 1:** **Top ten SNPs by population- and family-based screening aggregation**.

**Power rank**	**SNP**	**CHR**	**BP**	**Minor Allele Freq**	**Fisher's *p*-value**	**METAL *p*-value**	**GAP *p*-value**	**FBAT *p*-value**	**Singletons only *p*-value**	**Pooled cases/controls *p*-value**
1	rs11645147	16	9802457	0.377	1.74E-04	3.28E-07	3.09E-06	1.16E-03	5.71E-05	7.93E-04
2	rs7866522	9	8812704	0.284	3.28E-03	3.02E-03	5.61E-03	3.95E-01	1.10E-04	1.26E-01
3	rs847986	7	12454877	0.089	4.98E-02	6.81E-03	5.53E-03	2.71E-01	1.14E-03	3.52E-02
4	rs980519	4	72180823	0.065	8.42E-01	1.27E-01	5.90E-02	8.63E-01	1.23E-01	2.47E-03
5	rs11673097	19	57119434	0.293	3.25E-02	3.80E-04	1.50E-03	8.38E-01	2.77E-05	2.85E-03
6	rs4707991	6	73493822	0.332	6.78E-01	1.00E-01	1.28E-01	8.81E-01	7.15E-02	1.13E-03
7	rs17689531	4	72022775	0.120	8.81E-01	9.33E-01	9.15E-01	9.23E-01	9.59E-01	9.26E-02
8	rs1901712	4	72147303	0.065	9.25E-01	9.37E-02	3.70E-02	6.38E-01	1.08E-01	2.01E-03
9	rs17470789	5	144584265	0.125	5.88E-02	2.72E-01	1.78E-01	1.86E-02	8.41E-01	5.92E-01
10	rs8179278	1	234379913	0.091	5.01E-02	5.19E-05	5.25E-05	8.67E-01	2.18E-05	2.66E-03

Aggregated SNP rankings based on conditional power for family-based and population-based studies were calculated. The top ten ranked SNPs were selected per convention to minimize the need for multiple comparison correction (0.05/10 = 0.005). Fisher's combined p-values for diabetic nephropathy trait association were obtained using the corresponding association method used for power analysis from FBAT and C2BAT. METAL and Genetic Association analysis Platform (GAP) aggregated p-values were also obtained but are subjected to genome-wide multiple comparison correction (0.05 /384,197 = 1.3 × 10^−7^). Genome-wide family-based association testing (FBAT) included trio probands and parents. Cochran-Armitage test for trend for an additive genetic model adjusted for sex and stratified by center ascertainment using Cochran-Mantel-Haenszel method were obtained using unrelated cases and controls only (singletons only) and by combining cases and controls from unrelated subjects and trio probands (pooled cases and controls).

We sought to replicate the two genome-wide significant SNPs using the Family Investigation of Nephropathy and Diabetes (FIND) study (Knowler et al., 2005; Iyengar et al., 2007; Igo et al., 2011). The FIND study recruited diabetic subjects with and without nephropathy. Most FIND participants with GWAS have type 2 diabetes (between 90 and 95%), and the majority of nephropathy controls used in this sub-study are relatives of index cases. To be consistent with the GoKinD population, we examined only European American subjects. Rs11645147 conferred a *p*-value of 0.004 assuming a dominant mode of inheritance; rs7866522 failed to reach significance. While FIND shares the nephrotic phenotype with GoKinD, it includes primarily type 2 diabetics as opposed to type 1, making comparisons inexact. In addition, the dominant mode of inheritance was the only one for which rs11545147 garnered nominal significance, although it still reached significance after adjusting for testing multiple modes of inheritance.

## Discussion

The primary finding of this study is that analysis of GoKinD collection by any of a strict population-based design, a family-based design or the combined approach without any screening, did not detect genome-wide significant SNPs. Simply combining family-based association results with those from population-based data actually suppressed areas of suggestive genome-wide significance compared to the original GoKinD GWAS, possibly by correcting for previously unrecognized population substructure; however, the definitive reason for this is unknown. Conversely, the incorporation of family-based information also uncovered new areas of possible interest. Two SNPs reached significance in our combined data analysis by the novel two-step approach using Van Steen screening with the family-based trios and C2BAT data partitioning in the unrelated case-control data, which ranks markers by conditional power and then selects the top 10 overall ranked markers.

Suggestive findings using only population-based association tests with all unrelated cases and controls, when pooled with trio probands as in the Pezzolesi study, were not replicated by either the family-based or combined analyses. This finding could suggest the presence of unresolved population structure despite using PCA to select a homogenous population, and that earlier suggestive SNPs were likely false positive associations. It also could be a result of a decrease in power from using family-based tests. This balance of increased robustness against problems of population stratification and a decrease in power are common factors when considering family-based tests.

Compared to analyzing either of the unrelated case-control or trio datasets alone, the additional sample size via the combined Fisher's method increases study power, and this may explain the new areas of suggestive significance. The lack of findings of genome-wide significant SNPs may reflect that there are truly no associations between DN and genotyped markers among the GoKinD dataset or that the study reflects the difficulties encountered with the multiple testing problem inherent to GWAS.

Applying screening methods due to Van Steen et al. (2005) and McQueen et al. (http://rss.acs.unt.edu/Rdoc/library/pbatR/html/c2bat.html), statistically independent assessments of each SNP's power to detect an association allows for more efficient genome-wide testing. Here we aggregated the independently obtained marker rankings using parental information in the family-based data and data partitioning in the population-based data. By limiting testing to the conventionally-used top 10 highest ranked markers (Herbert et al., 2006), two SNPs reached genome-wide significance. While this result is appealing, without extensive simulation to establish operating characteristics of the suggested approach in other settings, caution must be taken to not over interpret. It does suggest, at the least, that future methodological work in this regard is warranted. We plan to investigate the performance of this approach in other scenarios and examine implications of varying the choice of the number of SNPs to carry to the testing stage as well as the function for rank aggregation.

With the growing availability of GWAS and now sequencing data, association studies have increasingly reported positive results. Multiple-hypothesis testing, low power, study design variability, phenotypic definition, and population structure continue to pose investigational difficulties (Laird and Lange, 2006). Family-based and population-based case control designs each have unique strengths and weaknesses, but when used in a complementary fashion as proposed, they may overcome these challenges.

### Conflict of interest statement

The authors declare that the research was conducted in the absence of any commercial or financial relationships that could be construed as a potential conflict of interest.

## Supplementary material

Figure S1 **Q-Q Plots for association within controls and cases**. When controls and cases from each center of ascertainment are combined by affection status, an over dispersion of the Cochran-Armitage test statistic for trend is noted. The deviation from expected, confirmed by an elevated genomic control inflation factor (λ_GC_> 1.05), suggests underlying confounding and stratification by center ascertainment between the Joslin Diabetes Center and the George Washington University Biostatistical Center.

Figure S2 **Mendelian errors per GoKinD family trio**. In the GoKinD family-based study, 551 trios (two parents and one offspring) were assessed for Mendelian errors of transmission. Number of Mendel SNP errors per family was log_10_ transformed. A single outlier family was determined by a greater than 5% Mendel error rate (>20,000 errors, indicated by the red arrow). Mendelian errors reflect poor quality SNP genotype calling, poor DNA sample quality or inconsistent familial relationship.

Figure S3 **Projection of principal components of population-based GoKinD subjects onto HapMap populations**. A pruned set of SNPs (85,051) from the population-based cases and controls were projected onto a similar set of SNPs from the original three International HapMap populations [GoKinD subjects in blue, **(A)**]. Using *Z*-scores based on median absolute deviation, a homogenous population was selected for association analysis [selected GoKinD population shown in blue and outliers in red, **(B)**]. HapMap populations: CEU (Eastern and Western European) samples are shown in green, YRI (Yoruba in Ibadan, Nigeria) are in black, and JPT + CHB (Japanese in Tokyo, Japan and Hans Chinese in Beijing) are shown in violet.

## Appendix

### Recruitment

Of the 1879 T1D subjects initially recruited, 10 failed genotyping on an Affymetrix 5.0 (500 K) SNP array conducted by the GAIN genotyping laboratory at the Broad Institute (Cambridge, MA) and the Central Biochemistry Laboratory at the University of Minnesota. Of the remaining 1869 subjects, 21 were excluded from the data release due to sample duplication detected by identifying cryptic relatedness and eight were removed due to assay plate failure via genotype calling interference (https://www.niddkrepository.org/GOKIND) (Pluzhnikov et al., 2010). Of the 1840 remaining, none were detected as cryptically related using identity-by-descent proportion estimation $\left(\hat{\pi }<0.1621\right)$.

GoKinD samples were recruited under two separate ascertainment protocols at JDC and GWU. Using Q-Q plots, over dispersion of the Cochran-Armitage test statistic for trend for JDC vs. GWU, among controls and cases, separately were demonstrated (Supplemental Figure 1). To test if the observed overall inflation factor (λ_GC_) (Devlin and Roeder, 1999) for cases (λ_GC_ = 1.097) and controls (λ_GC_ = 1.115) were truly significant for stratification, centers were permutated by affection status. For 1000 permutations in cases and controls, no λ_GC_ were more extreme (*p*-value < 10^−3^); hence further association testing was stratified by center.

### Singletons

Of the 1270 population-based singletons remaining, four were removed for sex mismatch and one for high individual genotype missingness (>0.10), which left 1265 (650 cases and 615 controls) (Supplemental Table 1).

### Trios

Of the 571 family-based trios, 551 included genotyping for both parents (full trios) while 20 included only a single founder. Three subjects and their parents were excluded for sex mismatch. Families were evaluated for Mendelian error rates to assess validity of relatedness and the degree of genotyping error: one was excluded with a Mendelian error rate greater than 5% (Supplemental Figure 2). This subject was added to the singletons but was excluded due to high individual genotype missingness, which confirms the original Mendelian error finding. A total of 567 parent(s)/offspring were included (264 case and 303 control offspring) (Supplemental Table 1).

### SNP quality control

For autosomal chromosomes, both population- and family-based SNPs were filtered for an overall minor allele frequency (MAF) <0.01, Hardy-Weinberg equilibrium probability = 1 × 10^−5^, duplicate SNPs, and sequential missingness by MAF; 95% overall minimum completeness, 97% for MAF between 5–10%, and 99% for infrequent SNPs with MAF between 1 and 5% (Burton et al., 2007; Ziegler et al., 2008; Pezzolesi et al., 2009). For population-based C2BAT power analysis, an overall MAF <0.05 screening was used per computational software restriction. In addition, family-based SNPs were filtered for a Mendelian error rate of 3 per SNP based on a subset of full trios excluding families with > 10,000 errors per family. A final 384,197 and 338,970 singleton SNPs (PLINK and C2BAT analysis, respectively) and 352,004 trio SNPs were analyzed (Supplemental Table 2).

### Population structure

To select a homogenous population in the singleton cases and controls, PCA was performed by projection of a pruned subset of SNPs (85,051) onto the three original HapMap populations [Utah residents with ancestry from northern and western Europe (CEU), Yoruba in Ibadan, Nigeria (YRI), Japanese in Tokyo, Japan and Hans Chinese in Beijing, China (JPT_CHB), http://pngu.mgh.harvard.edu/~purcell/plink/res.shtml#hapmap, Phase 2, release 23] (Gibbs et al., 2003) using EIGENSOFT (Patterson et al., 2006; Price et al., 2006) and EIGENSOFTplus (Weale, 2009, 2010) software. Singleton genotypes were pruned with PLINK's (Purcell et al., 2007) in-depth pairwise option (500 SNP sliding window, 5 SNP step), with additional removal of long range linkage disequilibrium areas (Supplemental Figure 3A). Outliers were determined using visual assessment and calculated *Z*-scores based on median absolute deviation, i.e., median (|X—median (X) |). Ninety-four subjects were excluded at a *Z*-score of 9.1 for a final singleton sample of 1171 (576 cases, 597 controls) (Supplemental Figure 3B).
