# Supplementary material for: Combining genetic association study designs: a GWAS case study
Source: Front Genet. 2013 Sep 27;4:186. doi: 10.3389/fgene.2013.00186 (PMC3784826; doi:10.3389/fgene.2013.00186)
Supplement: Figure S1 — Q-Q Plots for association within controls and cases. When controls and cases from each center of ascertainment are combined by affection status, an over dispersion of the Cochran-Armitage test statistic for trend is noted. The deviation from expected, confirmed by an elevated genomic control inflation factor (λGC> 1.05), suggests underlying confounding and stratification by center ascertainment between the Joslin Diabetes Center and the George Washington University Biostatistical Center. [file Data_Sheet_1.ZIP › Fardo/51204_Fardo_Supplementary_Table_S1.DOCX]

|  | | |  |  | |
| --- | --- | --- | --- | --- | --- |
|  | Population-based Subjects | | Family-based Subjects | | |
| Filter Criteria | Controls | Cases | Controls | | Cases |
| Total | 619 | 651 | 305 | | 266 |
| (Men/Women) | (251/368) | (347/304) | (125/180) | | (117/149) |
| Sex mismatched | 3 | 1 | 1 | | 2 |
| Genotype missingness > 0.1 | 1 |  |  | |  |
| Mendelian/family error > 5% |  |  | 1 | |  |
| Population Outlier | 21 | 73 |  | |  |
| (Men/Women) | 8/13 | 31/42 |  | |  |
| Post quality check | 594 | 577 | 303 | | 264 |
| (Men/Women) | (242/352) | (315/262) | (124/179) | | (117/147) |

**Supplemental Table 1.**  **Subject Quality Control Analysis.** Subject quality control analyses included screening for sex mismatching between phenotypic and genotypic data and overall genotyping missingness of greater than 10%. Family-based data allowed for assessment of Mendelian errors of greater than 5% within trios. Using principal component analysis (PCA), a homogenous population based on northern and western European ancestry was selected for unrelated population-based cases and controls.
