# Supplementary material for: Combining genetic association study designs: a GWAS case study
Source: Front Genet. 2013 Sep 27;4:186. doi: 10.3389/fgene.2013.00186 (PMC3784826; doi:10.3389/fgene.2013.00186)
Supplement: Figure S1 — Q-Q Plots for association within controls and cases. When controls and cases from each center of ascertainment are combined by affection status, an over dispersion of the Cochran-Armitage test statistic for trend is noted. The deviation from expected, confirmed by an elevated genomic control inflation factor (λGC> 1.05), suggests underlying confounding and stratification by center ascertainment between the Joslin Diabetes Center and the George Washington University Biostatistical Center. [file Data_Sheet_1.ZIP › Fardo/51204_Fardo_Supplementary_Table_S2.DOCX]

|  | | | | |
| --- | --- | --- | --- | --- |
|  | **Population-based Case/Control** | | **Family-based**  **Trios** | |
|  | Number Filtered | Total Remaining | Number Filtered | Total Remaining |
| Total genotyped |  | 469094 |  | 469094 |
| Overall Criteria: |  |  |  |  |
| Autosomal (Sex chromosomes excluded) | 9792 | 459302 | 9792 | 459302 |
| Mendel error ≥3 |  |  | 35610 | 423692 |
| HWE < 1e-5 | 1021 |  | 240 |  |
| MAF < 0.01 (SNPs may fail both criteria) | 49106 | 409219 | 57421 | 366118 |
| Sequential Filtering: |  |  |  |  |
| Missing ≥0.05 | 17643 |  | 9561 |  |
| Missing 0.05<*x*<0.03 and MAF 0.05≤*x*<0.10 | 1343 |  | 811 |  |
| Missing >0.01 and MAF <0.05 | 6035 |  | 3742 |  |
| Sequential filtering total: | 25021 | 384198 | 14114 | 352004 |
| Duplicate Marker | 1 | 384197 | 0 |  |

**Supplemental Table 2. SNP Quality Filter Results by Criteria Threshold.** Autosomal SNPs were filtered to excluded those with minor allele frequency < 1% and those out of Hardy-Weinberg equilibrium (p< 1x10^-5^). SNPs with greater than 3 Mendelian errors among complete family trios were excluded from family-based testing. Sequential filtering for SNP missingness was employed. Overall genotyping missingness for each SNP across subjects was limited to no more than 5%. As SNP minor allele frequency decreased (becoming more rare), missingness tolerance became more stringent.
