# Supplementary material for: Combining genetic association study designs: a GWAS case study
Source: Front Genet. 2013 Sep 27;4:186. doi: 10.3389/fgene.2013.00186 (PMC3784826; doi:10.3389/fgene.2013.00186)
Supplement: Figure S1 — Q-Q Plots for association within controls and cases. When controls and cases from each center of ascertainment are combined by affection status, an over dispersion of the Cochran-Armitage test statistic for trend is noted. The deviation from expected, confirmed by an elevated genomic control inflation factor (λGC> 1.05), suggests underlying confounding and stratification by center ascertainment between the Joslin Diabetes Center and the George Washington University Biostatistical Center. [file Data_Sheet_1.ZIP › Fardo/51204_Fardo_Supplementary_Table_S3.DOCX]

|  | | | | | | | | | | | | |
| --- | --- | --- | --- | --- | --- | --- | --- | --- | --- | --- | --- | --- |
| SNP | Chr | BP | Fisher's | Pooled Cases/ Controls | Singletons  Only | | FBAT | METAL | GAP | ScreenRank | MAF | Gene location or proximity |
|  |  |  | p-values | | | | | | |  |  | If suggestive significance noted |
| rs8179278 | 1 | 234379913 | 5.01E-02 | 2.66E-03 | 2.18E-05 | 8.67E-01 | | 5.19E-05 | 5.25E-05 | 10 | 0.091 |  |
| rs17689531 | 4 | 72022775 | 8.81E-01 | 9.26E-02 | 9.59E-01 | 9.23E-01 | | 9.33E-01 | 9.15E-01 | 7 | 0.120 |  |
| rs1901712 | 4 | 72147303 | 9.25E-01 | 2.01E-03 | 1.08E-01 | 6.38E-01 | | 9.37E-02 | 3.70E-02 | 8 | 0.065 |  |
| rs980519 | 4 | 72180823 | 8.42E-01 | 2.47E-03 | 1.23E-01 | 8.63E-01 | | 1.27E-01 | 5.90E-02 | 4 | 0.065 |  |
| rs981885 | 5 | 65629780 | 1.32E-02 | 1.95E-04 | 7.80E-04 | 1.80E-03 | | **7.79E-06** | 2.54E-05 |  | 0.367 | Unknown |
| rs1544933‡ /rs10471321 | 5 | 65640226 | 6.48E-04 | 3.35E-04 | 1.12E-03 | 9.77E-04 | | **7.60E-06** | 2.56E-05 |  | 0.383 | Unknown |
| rs17470789 | 5 | 144584265 | 5.88E-02 | 5.92E-01 | 8.41E-01 | 1.86E-02 | | 2.72E-01 | 1.78E-01 | 9 | 0.125 |  |
| rs1046089 | 6 | 31602967 | 2.40E-05 | 1.03E-01 | 2.16E-01 | **3.69E-06** | | 6.57E-04 | **1.10E-06** |  | 0.479 | BAT2 |
| rs805301 | 6 | 31618121 | 5.56E-04 | 7.95E-02 | 1.82E-01 | 1.15E-04 | | 1.76E-03 | **4.43E-06** |  | 0.455 | Unknown |
| rs2894249‡ /rs3132959 | 6 | 32325835 | 2.08E-05 | 4.87E-01 | 8.29E-01 | **1.44E-06** | | 6.26E-02 | 1.38E-05 |  | 0.119 | C6orf10 |
| rs3129932 | 6 | 32336127 | 2.45E-05 | 4.87E-01 | 8.29E-01 | **1.71E-06** | | 6.42E-02 | 1.47E-05 |  | 0.115 | C6orf10 |
| rs3135377 | 6 | 32385399 | **9.74E-06** | 9.63E-01 | 4.32E-01 | **9.74E-06** | | 2.77E-01 | 1.68E-05 |  | 0.090 | C6orf10 |
| rs9469220 | 6 | 32658310 | 1.92E-05 | 5.01E-01 | 3.44E-01 | **1.32E-06** | | 9.62E-02 | 5.43E-04 |  | 0.335 | HLA-DQB1 HLA-DQA2 |
| rs4707991 | 6 | 73493822 | 6.78E-01 | 1.13E-03 | 7.15E-02 | 8.81E-01 | | 1.00E-01 | 1.28E-01 | 6 | 0.332 |  |
| rs847986 | 7 | 12454877 | 4.98E-02 | 3.52E-02 | 1.14E-03 | 2.71E-01 | | 6.81E-03 | 5.53E-03 | 3 | 0.089 |  |
| rs2722292 | 7 | 37824581 | 2.00E-05 | 8.02E-04 | 5.93E-02 | **4.36E-06** | | 1.23E-04 | 1.96E-04 |  | 0.259 | GPR141 TXNDC3 |
| **rs7866522** | 9 | 8812704 | **3.28E-03** | 1.26E-01 | 1.10E-04 | 3.95E-01 | | 3.02E-03 | 5.61E-03 | 2 | 0.284 | Protein tyrosine phosphatase, receptor, D region (PTPRD) |
| rs10868025 | 9 | 86164176 | 7.36E-03 | **2.56E-06** | 3.85E-04 | 1.53E-02 | | 1.81E-05 | 3.50E-05 |  | 0.383 | FRMD3 C9orf103 |
| rs1450193 | 9 | 118731889 | 2.92E-01 | 2.91E-03 | **6.92E-06** | 2.92E-01 | | 3.09E-05 | 3.06E-03 |  | 0.026 | C9orf27 closest |
| rs11186286 | 10 | 92477076 | 5.66E-03 | **5.08E-06** | 1.30E-05 | 3.42E-01 | | 1.36E-05 | 2.82E-04 |  | 0.174 | SNRPD2P1 HTR7 |
| rs739401† /rs451041 | 11 | 3036324 | 1.47E-05 | 3.13E-05 | 8.08E-03 | **6.89E-06** | | **5.45E-06** | 5.11E-05 |  | 0.494 | CARS |
| rs1980089 | 11 | 72288725 | 1.16E-03 | 3.60E-03 | 2.72E-03 | 2.76E-03 | | 3.98E-05 | **9.58E-06** |  | 0.484 | Phosphodiesterase 2A, cGMP-stimulated |
| rs10842504 | 12 | 25353517 | 8.67E-03 | NA | **2.61E-06** | NA | | **2.61E-06** | 1.05E-04 |  | 0.489 | LYRM5 |
| rs7956328 | 12 | 125125465 | 6.52E-03 | **5.00E-06** | **5.44E-06** | 8.02E-01 | | 3.73E-05 | 1.82E-04 |  | 0.282 | NCOR2 SCARB1 |
| rs1041466 | 13 | 110244322 | 1.23E-02 | **1.34E-06** | 2.30E-05 | 1.17E-01 | | **8.88E-06** | 7.21E-05 |  | 0.445 | MYO16 IRS2 |
| rs1411766‡ /rs17412858 | 13 | 110252160 | 2.03E-02 | **6.51E-07** | 4.72E-05 | 2.64E-01 | | 4.42E-05 | 6.65E-05 |  | 0.348 | MYO16 IRS2 |
| rs6492208‡ /rs2391777 | 13 | 110257726 | 1.17E-02 | **2.44E-06** | 4.33E-05 | 1.40E-01 | | 1.90E-05 | 7.40E-05 |  | 0.406 | MYO16 IRS2 |
| rs9515085 | 13 | 110258782 | 3.71E-03 | **7.63E-06** | 1.79E-04 | 2.12E-01 | | 1.08E-04 | 8.63E-05 |  | 0.371 | MYO16 IRS2 |
| rs9521445δ /rs7989848 | 13 | 110285534 | 2.34E-02 | **1.88E-06** | 1.13E-04 | 2.65E-01 | | 9.77E-05 | 7.95E-05 |  | 0.479 | MYO16 IRS2 |
| rs10518756 | 15 | 42522792 | 4.72E-03 | 1.67E-04 | **2.83E-06** | 5.55E-01 | | 6.29E-05 | 1.21E-03 |  | 0.173 | Transmembrane protein 87A |
| **rs11645147** | 16 | 9802457 | **1.74E-04** | 7.93E-04 | 5.71E-05 | 1.16E-03 | | **3.28E-07** | **3.09E-06** | 1 | 0.377 | Glutamate [NMDA] receptor subunit (GRIN2A) |
| rs11673097 | 19 | 57119434 | 3.25E-02 | 2.85E-03 | 2.77E-05 | 8.38E-01 | | 3.80E-04 | 1.50E-03 | 5 | 0.293 | Zinc finger protein 71 (ZNF71) |
| ‡ In complete linkage disequilibrium (r2 = 1.0) | | | | |  |  | |  |  |  |  |  |
| †In high linkage disequilibrium (r2 = 0.983) | | | | |  |  | |  |  |  |  |  |
| δIn high linkage disequilibrium (r2 = 0.901) | | | | |  |  | |  |  |  |  |  |
| ns P-value > 1.00E-04 NA -SNP not assessed | | | | |  |  | |  |  |  |  |  |

**Supplemental Table 3. Top SNPs in the GoKinD study of diabetic nephropathy in type 1 diabetics across analytic methods.** SNPs of interest are displayed along with location, minor allele frequency and p-values across the methods explored. P-values of suggestive significance for each method are in **bold**.
