# Supplementary figures and images for: Combining genetic association study designs: a GWAS case study
Source: Front Genet. 2013 Sep 27;4:186. doi: 10.3389/fgene.2013.00186 (PMC3784826; doi:10.3389/fgene.2013.00186)

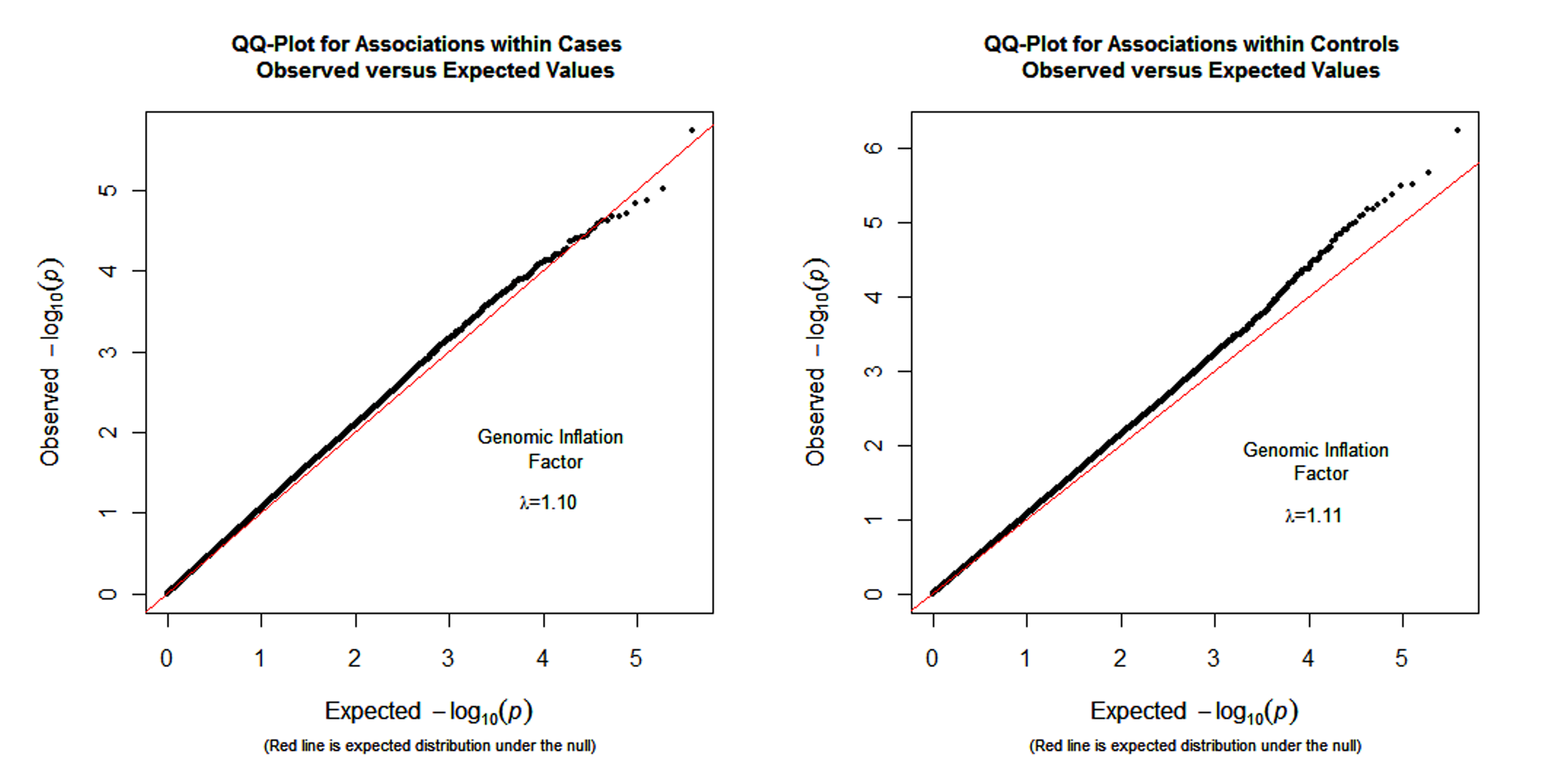

Supplement: Figure S1 — Q-Q Plots for association within controls and cases. When controls and cases from each center of ascertainment are combined by affection status, an over dispersion of the Cochran-Armitage test statistic for trend is noted. The deviation from expected, confirmed by an elevated genomic control inflation factor (λGC> 1.05), suggests underlying confounding and stratification by center ascertainment between the Joslin Diabetes Center and the George Washington University Biostatistical Center. [file Data_Sheet_1.ZIP › Fardo/S1.TIF]

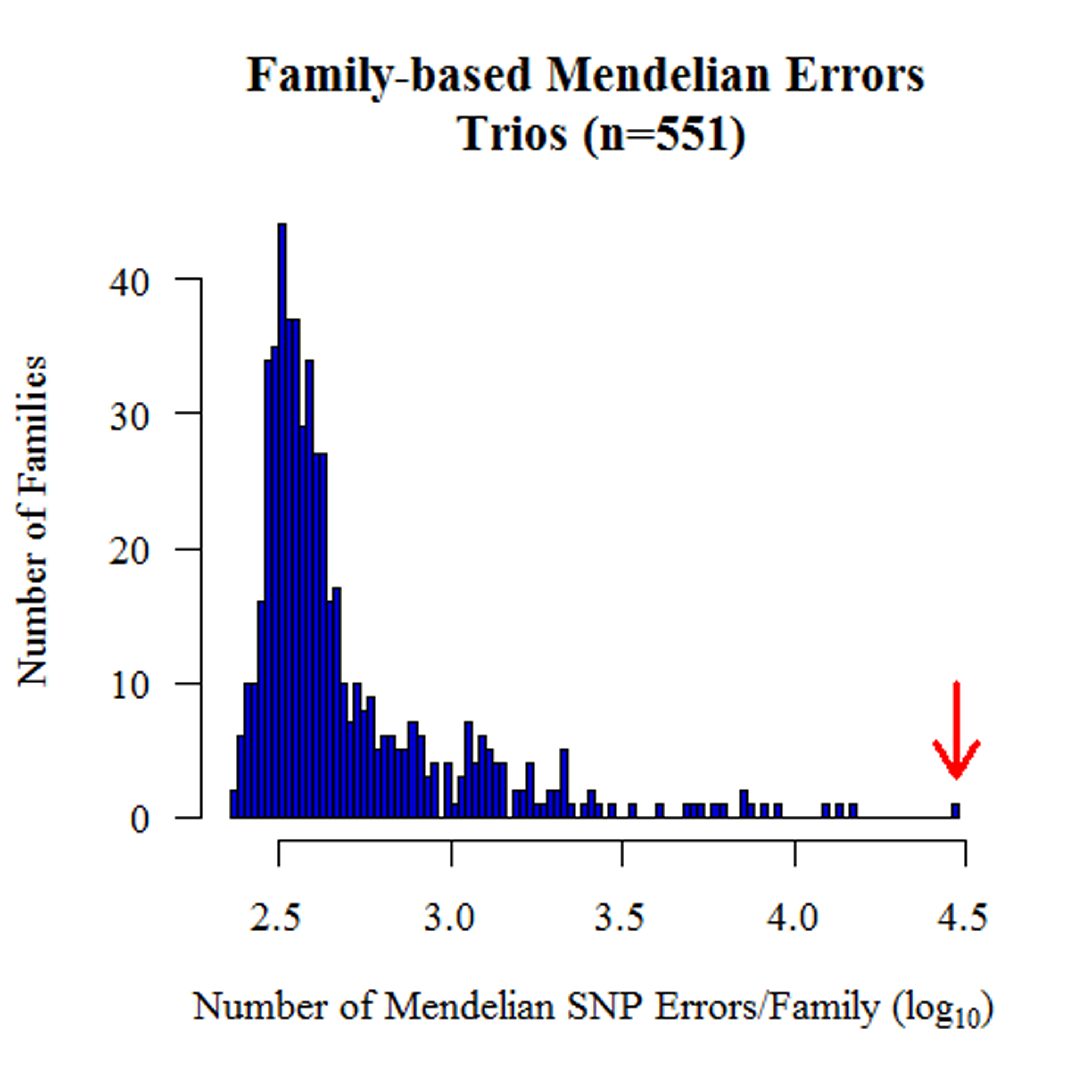

Supplement: Figure S1 — Q-Q Plots for association within controls and cases. When controls and cases from each center of ascertainment are combined by affection status, an over dispersion of the Cochran-Armitage test statistic for trend is noted. The deviation from expected, confirmed by an elevated genomic control inflation factor (λGC> 1.05), suggests underlying confounding and stratification by center ascertainment between the Joslin Diabetes Center and the George Washington University Biostatistical Center. [file Data_Sheet_1.ZIP › Fardo/S2.TIF]

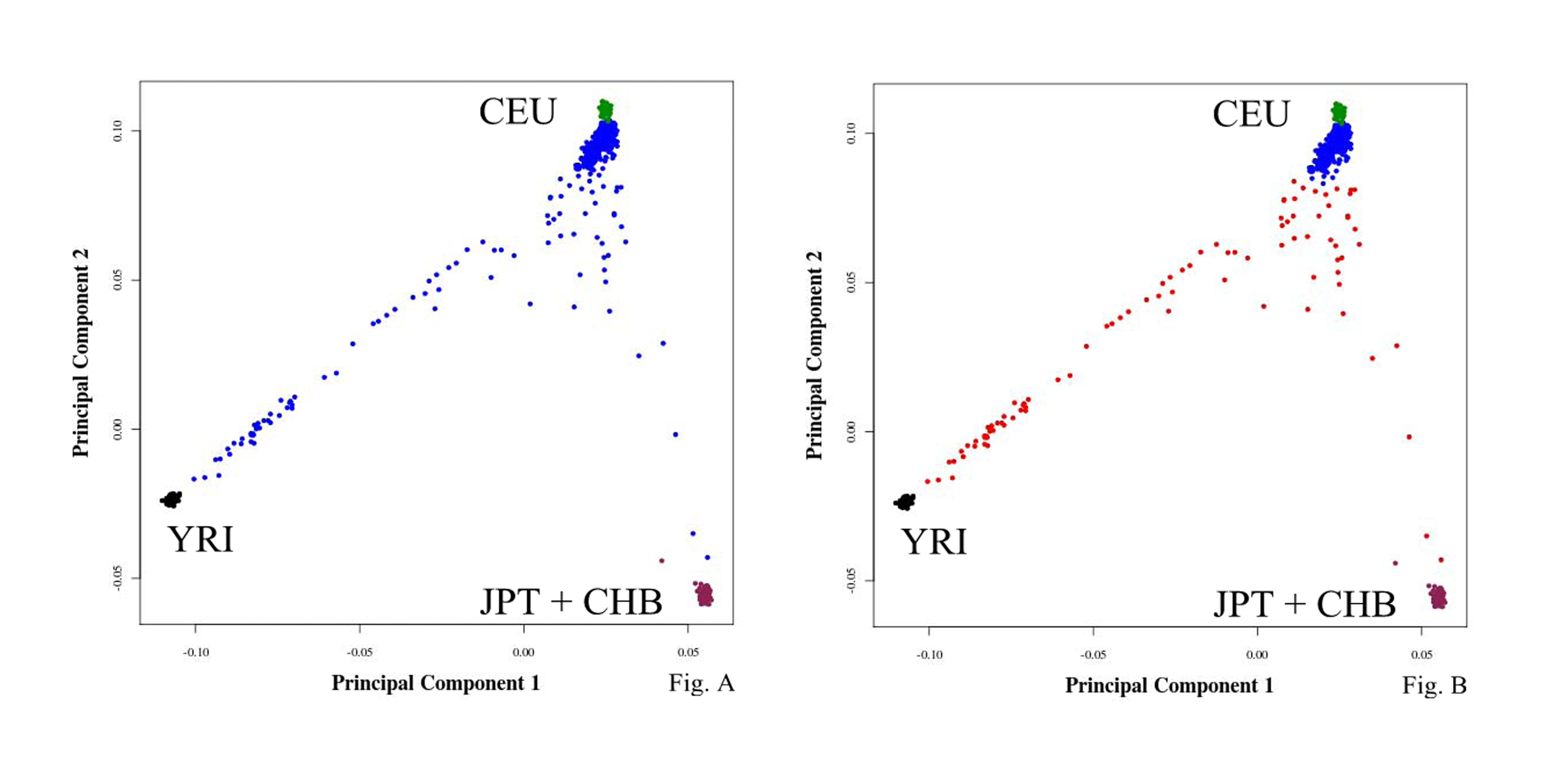

Supplement: Figure S1 — Q-Q Plots for association within controls and cases. When controls and cases from each center of ascertainment are combined by affection status, an over dispersion of the Cochran-Armitage test statistic for trend is noted. The deviation from expected, confirmed by an elevated genomic control inflation factor (λGC> 1.05), suggests underlying confounding and stratification by center ascertainment between the Joslin Diabetes Center and the George Washington University Biostatistical Center. [file Data_Sheet_1.ZIP › Fardo/S3.TIF]
